# Macrophage Migration Inhibitory Factor Activates Hypoxia-Inducible Factor in a p53-Dependent Manner

**DOI:** 10.1371/journal.pone.0002215

**Published:** 2008-05-21

**Authors:** Seiko Oda, Tomoyuki Oda, Kenichiro Nishi, Satoshi Takabuchi, Takuhiko Wakamatsu, Tomoharu Tanaka, Takehiko Adachi, Kazuhiko Fukuda, Gregg L. Semenza, Kiichi Hirota

**Affiliations:** 1 Department of Anesthesia, Kyoto University Hospital, Kyoto University, Kyoto, Japan; 2 The Tazuke Kofukai Medical Research Institute Kitano Hospital, Osaka, Japan; 3 Department of Cardiovascular Medicine, Graduate School of Medicine, Kyoto University, Kyoto, Japan; 4 Department of Anesthesiology, Kansai Medical University, Moriguchi-City, Osaka, Japan; 5 Vascular Biology Program, Institute for Cell Engineering, The Johns Hopkins University School of Medicine, Baltimore, Maryland, United States of America; University of Arkansas, United States of America

## Abstract

**Background:**

Macrophage migration inhibitory factor (MIF) is not only a cytokine which has a critical role in several inflammatory conditions but also has endocrine and enzymatic functions. MIF is identified as an intracellular signaling molecule and is implicated in the process of tumor progression, and also strongly enhances neovascularization. Overexpression of MIF has been observed in tumors from various organs. MIF is one of the genes induced by hypoxia in an hypoxia-inducible factor 1 (HIF-1)-dependent manner.

**Methods/Principal Findings:**

The effect of MIF on HIF-1 activity was investigated in human breast cancer MCF-7 and MDA-MB-231 cells, and osteosarcoma Saos-2 cells. We demonstrate that intracellular overexpression or extracellular administration of MIF enhances activation of HIF-1 under hypoxic conditions in MCF-7 cells. Mutagenesis analysis of MIF and knockdown of 53 demonstrates that the activation is not dependent on redox activity of MIF but on wild-type p53. We also indicate that the MIF receptor CD74 is involved in HIF-1 activation by MIF at least when MIF is administrated extracellularly.

**Conclusion/Significance:**

MIF regulates HIF-1 activity in a p53-dependent manner. In addition to MIF's potent effects on the immune system, MIF is linked to fundamental processes conferring cell proliferation, cell survival, angiogenesis, and tumor invasiveness. This functional interdependence between MIF and HIF-1α protein stabilization and transactivation activity provide a molecular mechanism for promotion of tumorigenesis by MIF.

## Introduction

Macrophage migration inhibitory factor (MIF) was originally described as a T cell–derived lymphokine but is now recognized as a pluripotent cytokine involved in multiple functions within and beyond the immune system [1]. In addition to its original ability to inhibit migration of macrophages, MIF exhibits a broad range of immunostimulatory and proinflammatory activities [2]. MIF is also produced by a variety of mesenchymal, parenchymal, and epithelial cell types, indicating its potential beyond the immune system. A number of studies have shown that MIF participates in the regulation of cell proliferation and differentiation and that it plays a role in the progress of septic shock, chronic inflammation, tissue damage, and autoimmune disease. Increased MIF mRNA expression has been reported in metastatic prostate cancer and ductal breast carcinoma. Following these findings, it became evident that MIF is involved in the development of lymphoma and tumor-associated angiogenesis [3], [4]. Inflammation is a critical component of tumor progression [5]. Many cancers arise from sites of infection, chronic irritation or inflammation. It is now becoming clear that the tumor microenvironment, which is strongly influenced by inflammatory cells, plays a major role in the neoplastic process, fostering proliferation, survival and migration of cancer cells. MIF represents an important link between inflammation and cancer [6], [7].

In response to hypoxia, dramatic changes in gene expression occur leading to increased synthesis of proteins such as erythropoietin (EPO), glucose transporters (GLUT), glycolytic enzymes, vascular endothelial growth factor (VEGF), and matrix metalloproteinases, which mediate cellular and tissue adaptation. The changes in gene expression are controlled by the transcriptional activator hypoxia-inducible factor 1 (HIF-1). HIF-1 was identified and purified as a nuclear factor that was induced in hypoxic cells and bound to the *cis*-acting hypoxia response element (HRE) located in the 3′-flanking region of the human *EPO* gene, which encodes erythropoietin [8]. HIF-1 is a heterodimeric protein that is composed of HIF-1α and HIF-1® subunits. Both HIF-1 subunits are members of the basic helix-loop-helix-containing PER-ARNT-SIM-domain family of transcription factors [8], [9].

The regulation of cellular HIF-1 activity occurs at multiple levels [10], [11]. The mechanisms regulating HIF-1α protein levels and transcriptional activity have been extensively analyzed. Two proline residues of HIF-1α are subjected to O_2_-dependent hydroxylation, which is required for binding of the von Hippel-Lindau tumor-suppressor protein (VHL), which is the recognition component of a ubiquitin-protein ligase that targets HIF-1α for rapid proteasomal degradation in non-hypoxic cells [12]. Under hypoxic conditions, the hydroxylation of proline-402 and proline-564 of HIF-1α is inhibited due to substrate (O_2_) limitation, resulting in HIF-1α protein stabilization [13]. The transcriptional activity of HIF-1α is also negatively regulated under normoxic conditions by hydroxylation of asparagine-803, which prevents interaction of HIF-1α with the coactivators p300 and CBP, thus blocking transcriptional activation [14]. The prolyl and asparaginyl hydroxylases utilize O_2_ and α-ketoglutarate as substrates [13], [14]. In contrast, HIF-1β is constitutively expressed in most cell types. Immunohistochemical analysis of human tumor biopsies has revealed overexpression of HIF-1α in various types of cancer [15]. High HIF-1α protein expression in tumors reflects stabilization of HIF-1α protein due to the frequent presence of intratumoral hypoxia as well as increased synthesis of the protein caused by genetic alterations involving oncogenes (such as HER2/neu) and tumor suppressor genes (such as p53, PTEN, and VHL) and autocrine growth factor signaling in cancer cells [16], [17]. HIF-1 activity is also induced by the stimulation of receptor tyrosine kinases or G protein-coupled receptors in cancer cells. HER2/neu activation increases the rate of HIF-1α protein synthesis via phosphatidylinositol 3-kinase (PI3K) and the downstream serine-threonine kinases AKT (protein kinase B) and mammalian target of rapamycin (mTOR). IGF-1-induced HIF-1α synthesis is dependent upon both the PI3K and MAP Kinase (MAPK) pathways.

Both MIF and HIF-1 play critical roles in cancer and inflammation [18]. Moreover, MIF has been identified as a hypoxia-induced gene in cancer cells [19], [20]. Based on these findings we investigated the functional relationship between MIF and HIF-1 in human cancer cells. In this study, we demonstrate that intracellular overexpression or extracellular administration of MIF enhances activation of HIF-1 under hypoxic conditions and that the regulation is not dependent on redox activity of MIF but on wild-type p53.

## Results

### MIF induces HIF-1α protein expression under hypoxic conditions in MCF-7 cells

**Figure 1:**
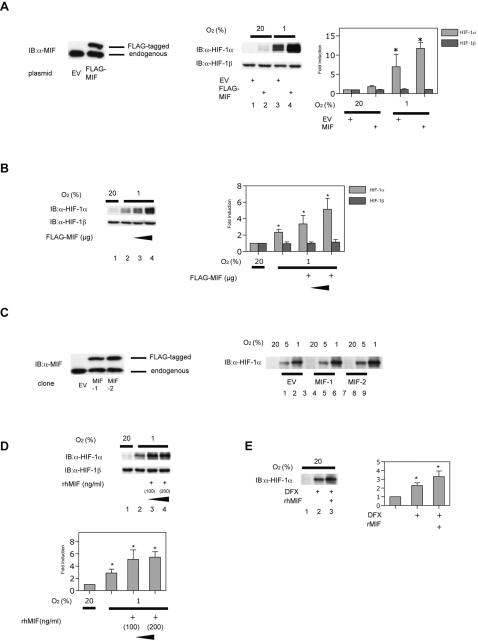
Effect of MIF on HIF-1 protein expression in MCF-7 cells. (A and B) MCF-7 cells were transfected with 2 µg of p3xFLAG-CMV-10 plasmid (EV) or pFLAG-MIF plasmids (A and B lane 4; 2 µg, B lane 3; 0.5 µg). (C) MCF-7 cells were transfected by the plasmids for expression of FLAG tag (EV) and FLAG-tagged MIF and selected by puromycin resistance. Two clones of MCF-7 cells stably expressing FLAG tag (EV) and FLAG-tagged MIF (MIF#1 and MIF#2 cells) were established. (D) MCF-7 cells were treated with the indicated doses of bacterially produced recombinant human (rh)MIF. (E) MCF-7 cells were treated with rhMIF. Cells were exposed to 20% (A, B, C, and D), 5% (C), or 1% (A, B, C, and D) O_2_ conditions or treated with 100 µM DFX (E) for 4 h. Cell were harvested for immunoblot assay for MIF (A and C), HIF-1α and HIF-1β protein (A, B, C, D, and E). Representative immunoblots are shown. Intensity of respective bands were analyzed densitometrically and fold induction to respective controls (lane1) are plotted accordingly as mean±S.D. (n = 3) *p<0.05 compared to 20% O_2_ conditions without MIF (lane 1) (ANOVA).

To investigate the impact of MIF on HIF-1 activity, the effect of intracellular transient overexpression of human MIF on HIF-1 protein expression was investigated. MCF-7 cells were transiently transfected with the expression vector pCMV-3xFLAG-hMIF or its empty vector pCMV-3xFLAG for 8 h. pCMV-3xFLAG-MIF significantly increased MIF protein expression (Fig. 1A, left panel). Cells were exposed to 20% or 1% O_2_ conditions for 4 h and then harvested for Western blotting analysis using anti-HIF-1α and HIF-1β antibodies. HIF-1α protein expression was induced by hypoxic treatment (Fig. 1A, right panel, lane 3) and furthermore increased by MIF (lane 4). Expression of HIF-1α under 20% O_2_ conditions was not significantly affected by MIF overexpression. HIF-1β protein expression was not affected by transient MIF overexpression under 1% or 20% O_2_ conditions (Fig. 1A, right panel). The effect of MIF overexpression on HIF-1α levels was dose-dependent (Fig. 1B).

We also investigated the effect of MIF using MCF-7 cells that stably expressed MIF. In two independent MIF-overexpressing clones (Fig. 1C, left panel), HIF-1α protein expression was induced more than control cells (lanes 2 and 3) under 5% (lanes 5 and 8) and 1% (lanes 6 and 9) O_2_ (Fig. 1C, right panel). HIF-1α expression was unchanged under 20% O_2_ regardless of the MIF expression level. HIF-1β protein expression was not affected by MIF (data not shown).

Because MIF is a cellular factor released from cells, we next investigated the impact of recombinant human MIF protein (rhMIF) on MCF-7 cells. rhMIF increased HIF-1α expression in a dose-dependent manner under hypoxic conditions whereas HIF-1β expression was not affected (Fig. 1D). rhMIF treatment also augmented HIF-1α protein levels in cells treated with iron chelator DFX which inhibits HIF-1α-hydroxylase activity.

### MIF induces HIF-1-dependent gene expression in MCF-7 cells

**Figure 2:**
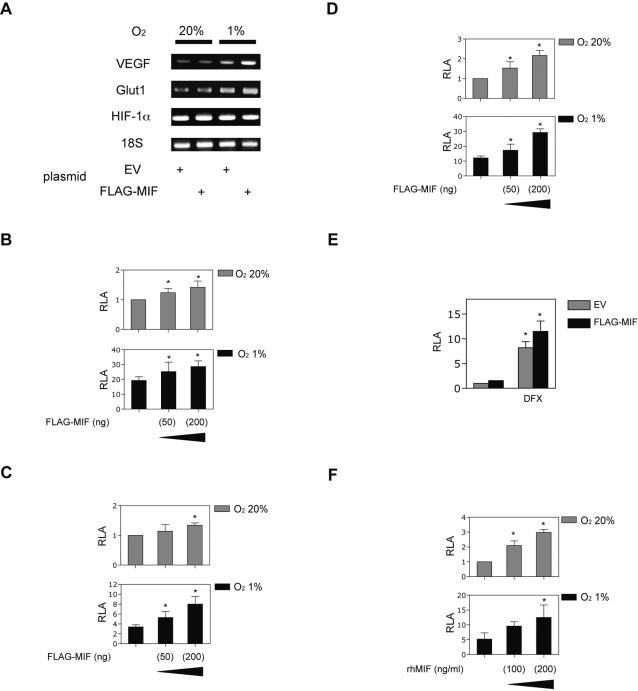
Effect of MIF on HIF-1-dependent gene expressions. (A) MCF-7 cells were transfected with the indicated plasmids and exposed to 20% or 1% O_2_ for 12 h. Then total RNA was isolated. Expression of VEGF, GLUT1, HIF-1α, and 18S rRNA was analyzed by RT-PCR using specific primer pairs. (B, C, D, and E) MCF-7 cells were transfected with pRL-SV40 encoding *Renilla* luciferase, FLAG-tagged MIF expression vector (B, C, and E), and one of the following plasmids encoding firefly luciferase: HRE reporter p2.1 (*B, C, and E*) or *VEGF* promoter reporter pVEGF-KpnI-Luc (*D*). (F) Constructs encoding the GAL4 DNA-binding domain (amino acids 1–147) fused to the indicated amino acids of HIF-1α were analyzed for their ability to transactivate reporter gene GAL4E1bLuc containing five GAL4-binding sites. MCF-7 cells were co-transfected with pRL-SV40 (50 ng), GAL4E1bLuc (100 ng), GAL4-HIF-1α fusion protein expression plasmids (100 ng), and FLAG-tagged MIF expression vector or empty vector (EV) (200 ng). Cells were treated with 100 µM of DFX or the indicated dose of rhMIF (F) and exposed to 20% or 1% O_2_ conditions for 18 h and then harvested. Results shown represent mean±S.D. of three independent transfections. *; *p*<0.05 compared to respective controls without MIF treatment (ANOVA).

We investigated whether MIF affected gene expression downstream of HIF-1 by RT-PCR analysis. MIF was transiently overexpressed in MCF-7 cells, which were exposed to 20% and 1% O_2_ for 18 h. As shown in Fig. 2A, VEGF and GLUT1 mRNA expression was induced by exposure to 1% O_2_ (1.73 and 1.77 fold, respectively). The induction of VEGF and GLUT1 mRNA was enhanced in hypoxic MIF-overexpressing MCF-7 cells (2.65 and 2.15 fold, respectively). Expression of HIF-1α mRNA was not affected by hypoxic or MIF treatment (Fig. 2A).

To determine whether the increase in VEGF and GLUT1 mRNA levels reflected an increase in HIF-1 transcriptional activity, MCF-7 cells were transfected with empty vector or FLAG-tagged MIF expression vector and the HRE-containing reporter plasmid p2.1. Overexpression of MIF significantly increased HRE-dependent gene expression significantly even under normoxic conditions and under hypoxic conditions in a MIF expression plasmid-dose dependent manner (Fig. 2B). Exposure of cells to bacterially produced rhMIF in the culture media also induced HRE-dependent gene expression under normoxic and hypoxic conditions (Fig. 2C). MIF also induced dose-dependent transcription of a luciferase reporter gene containing the intact human *VEGF* gene promoter encompassing nucleotides −2274 to +379 relative to the transcription start site (Fig. 2D). Overexpression of MIF also potentiated HRE-dependent gene expression induced by DFX (Fig. 2E).

Next, we investigated the impact of MIF on HIF-1α transcriptional activity. There are two independent transactivation domains (TADs) present in HIF-1α, which are designated as the amino-terminal (amino acids 531–575) and carboxyl-terminal (amino acids 786–826) TADs. A fusion protein consisting of the GAL4 DNA-binding domain fused to HIF-1α residues 531–826, which contains both TADs, is expressed at similar levels under hypoxic and non-hypoxic conditions and thus can be used to examine the transcriptional activity of HIF-1α independent of its protein expression [21]. rhMIF treatment increased transactivation mediated by GAL4-HIF-1α(531–826) in a dose-dependent manner (Fig. 2F) under both 20% and 1% O_2_ conditions. Thus, MIF not only increased HIF-1α protein levels but also the transcriptional activity of HIF-1α.

### MIF and TRX differentially regulate HIF-1 activity in MCF-7 cells

**Figure 3:**
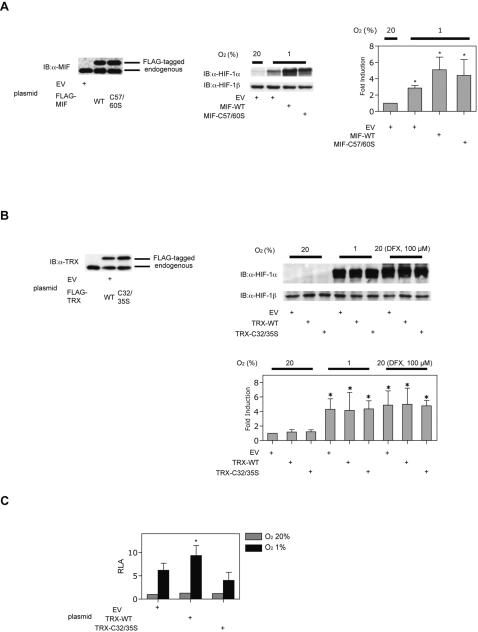
Effect of mutation of MIF on HIF-1 activity. (A and B) MCF-7 cells were transfected with 2 µg of p3xFLAG-CMV-10 plasmid, pFLAG-MIF-wt, or pFLAG-MIF-C57/60S plasmids (A) or p3xFLAG-CMV-10 plasmid, pFLAG-TRX-wt, or pFLAG-TRX-C32/35S plasmids (B). Cell were harvested for immunoblot assay for MIF (left panel), HIF-1α and HIF-1β protein (right panel). Experiments are repeated for three times. Representative immunoblots are shown. Intensity of respective bands were analyzed densitometrically and fold induction to lane 1 are plotted accordingly as mean±S.D. *p<0.05 compared to 20% O_2_ conditions without MIF treatment. (C) MCF-7 cells were transfected with p2.1 reporter plasmid, pRL-SV40 encoding *Renilla* luciferase and FLAG-tagged TRX-wt or −C32/35S expression vector. Cells were exposed to 20% or 1% O_2_ conditions for 18 h and then harvested. The luciferase activity of the lysates was determined using the Dual-Luciferase Reporter Assay System (Promega). The ratio of firefly to *Renilla* luciferase activity was determined. A normalized mean count±S.D. of three independent transfections is shown as relative luciferase activity (RLA). *; *p*<0.05 compared to respective control (ANOVA).

Because MIF is also an enzyme that exhibits oxidoreductase activity based on cysteine thiol-mediated mechanism [22]–[24], we next explored a possibility that the effect of MIF on HIF-1α is dependent on its redox activity. Wild-type MIF (MIF-wt) or its redox mutant MIF-C57/60S expression plasmid, which lacks oxidoreductase activity, was introduced transiently into MCF-7 cells (Fig. 3A, left panel) Cells were exposed to 20% or 1% O_2_ and cell lysates were subjected to immunoblot assays. The redox mutant MIF-C57/60S also increased HIF-1α protein expression in either transiently (Fig. 3A, right panel) or stably MIF-expressing cells (data not shown). HIF-1β expression was not affected by MIF-wt or MIF-C57/60S (Fig. 3A, right panel).

We next examined the effect of TRX, which also has thiol-reducing activity and shares a similar catalytic center with MIF. The wild-type TRX (TRX-wt) expression plasmid pCMV-3xFLAG-TRX-wt or its redox inactive mutant pCMV-3xFLAG-TRX-C32/35S was introduced transiently into MCF-7 cells (Fig. 3B, left panel). Expression of wild-type or mutant- TRX did not affect the baseline expression or hypoxia- or DFX-induced HIF-1α protein levels (Fig. 3B, right panel). In addition, addition of rhTRX to the culture media did not affect expression of HIF-1α (data not shown). HIF-1β expression was not affected by TRX (Fig. 3B, right panel). In contrast, expression of TRX-wt augmented, whereas TRX-C32/35S suppressed, hypoxia-induced HRE-dependent gene expression (Fig. 3C), as reported previously in HeLa cells [25].

**Figure 4:**
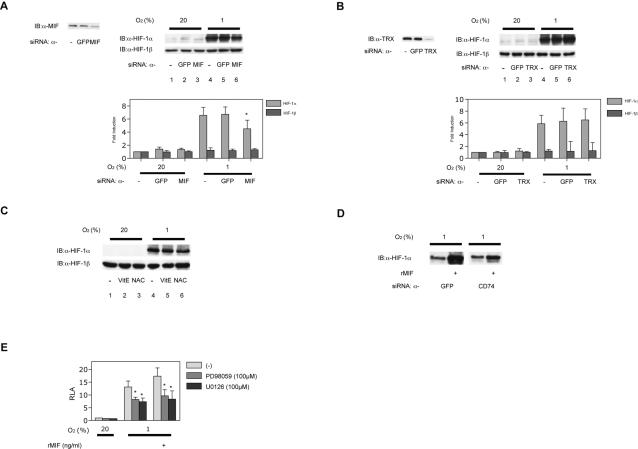
Effect of MIF-gene silencing on HIF-1 activity. (A, B, and D) MCF-7 cells were transfected with siRNA against MIF, TRX, CD74, or green fluorescence protein (GFP) as a negative control. Cells were exposed to 20% or 1% O_2_ conditions and were harvested for immunoblot assay for MIF or TRX (left panels) or HIF-1α and HIF-1β protein expression (right panels). Representative immunoblots are shown. Intensity of respective bands were analyzed densitometrically and fold induction to lane 1 are plotted accordingly as mean±S.D. (n = 3) (A, B, and C). *p<0.05 compared to 20% O_2_ conditions without treatment (ANOVA). (C) MCF-7 cells were pre-treated with 10 µM α-tocopherol or 10 mM NAC and then exposed to 20% or 1% O_2_ conditions for 4 h. Cells were harvested for immunoblot assay for HIF-1α and HIF-1β protein. (D) siRNAs for GFP or CD74 were introduced into MCF-7 cells. The cells were exposed to 1% O_2_ with or without rhMIF treatment. Cells were harvested for immunoblot assay for MIF HIF-1α protein. (E) MCF-7 cells were transfected with HRE reporter p2.1 and pRL-SV40 encoding *Renilla* luciferase. Cells were treated with the indicated dose of rhMIF and exposed to 20% or 1% O_2_ conditions for 18 h and then harvested. Results shown represent mean±S.D. of three independent transfections. *; *p*<0.05 compared to respective controls (ANOVA).

To investigate the effect of loss of function of MIF or TRX on HIF-1α protein levels, siRNA for MIF or TRX was introduced into MCF-7 cells. Protein expression of MIF or TRX was significantly suppressed by introduction of specific siRNAs (Fig. 4A and B, left panels). Under 1% O_2_ conditions, hypoxia-induced HIF-1α protein expression was inhibited by suppression of MIF but not that of TRX. In contrast, under 20% O_2_ conditions, expression of HIF-1α was not affected by knockdown of either MIF or TRX (Fig. 4A and B, right panel). Finally, MCF-7 cells were treated with antioxidants. Vitamin E or *N*-acetyl cysteine treatment did not affect HIF-1α or HIF-1β protein expression in MCF-7 cells under 20% or 1% O_2_ conditions (Fig. 4C).

### The effects of MIF is dependent on CD74 and ERK activity

We examined effect of interfering with the high-affinity binding protein for MIF CD74 mRNA expression [26]. siRNAs for GFP or CD74 were introduced into MCF-7 cells. The cells were exposed to 1% O_2_ with or without rhMIF treatment. Knockdown of CD74 expression abrogated effect of MIF over HIF-1α protein accumulation under 1% O_2_ conditions (Fig. 4D).

Because it was reported that MIF induced angiogenesis in a MAPK-dependent manner [27], we examined involvement of MAPK in MIF-elicited HIF-1 activation. Treatment with the MEK inhibitor PD98059 or U0126 suppressed MIF-induced HRE-dependent gene expression under 1% O_2_ conditions (Fig. 4E).

### The effect of MIF is not dependent on Jab1/CSN5 in MCF-7 cells

**Figure 5:**
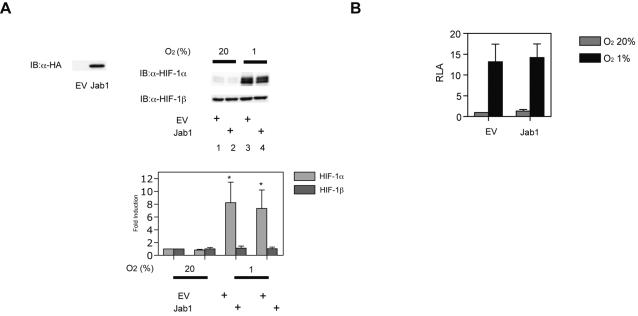
Involvement of Jab1 in MIF-induced HIF-1 activation. (A) MCF-7 cells were transfected with pSRα-HA-1 or pSRα-HA-Jab1. Cells were exposed to 20% or 1% O_2_ conditions and were harvested for immunoblot assay for Jab1 (left panels) or HIF-1α and HIF-1β protein expression (right panels). Representative immunoblots are shown. Intensity of respective bands were analyzed densitometrically and fold induction to lane 1 are plotted accordingly as mean±S.D. *p<0.05 compared to 20% O_2_ conditions without Jab1 (*Student t*-test). (B) MCF-7 cells were transfected with p2.1 reporter plasmid, pRL-SV40 encoding *Renilla* luciferase and pSRα-HA-1 or pSRα-HA-Jab1 expression vector. Cells were exposed to 20% or 1% O_2_ conditions for 18 h and then harvested. The luciferase activity of the lysates was determined using the Dual-Luciferase Reporter Assay System (Promega). The ratio of firefly to *Renilla* luciferase activity was determined. A normalized mean count±S.D. of three independent transfections is shown as relative luciferase activity (RLA).

Bae et al. reported that Jab1 (coactivator of c-Jun, also known as the CSN5 subunit of the COP9 signalosome) interfered with the binding of HIF-1α to the p53 tumor suppressor protein and thereby increased HIF-1α protein levels by enhancing HIF-1α stability in HEK293 cells [28]. Because MIF interacts with Jab1 and negatively regulates Jab1-controlled pathways [29], we investigated whether MIF-induced HIF-1 activation is dependent on Jab1. However, we failed to detect any effect of Jab1/CSN5 on HIF-1α protein expression (Fig. 5A) and HRE-dependent gene expression (Fig. 5B) in MCF-7 cells under either 20% or 1% O_2_ conditions.

### The effect of MIF is dependent on p53

**Figure 6:**
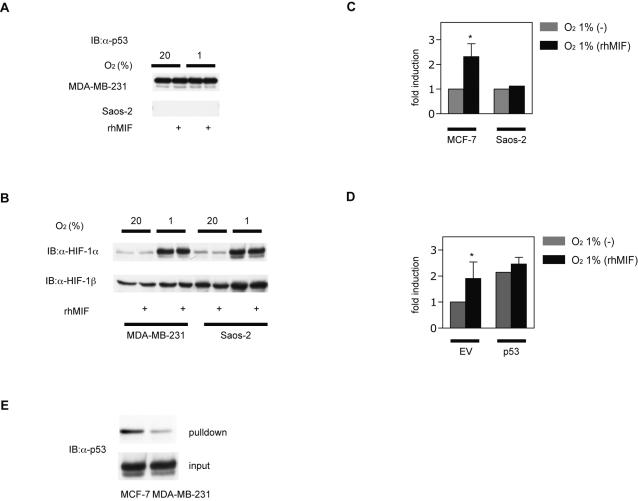
Involvement of p53 in MIF-induced HIF-1 activation. (A and B) MDA-MB-231 cells and Saos-2 cells were exposed to 20% or 1% O_2_ conditions with or without rhMIF treatment for 4 h and then harvested for immunoblot assay for p53 (A) and HIF-1α and HIF-1β protein (B). (C and D) MCF-7 cells and Saos-2 cells were transfected with p2.1 reporter and pRL-SV40 (C). MCF-7 cells transfected with pSUPER (EV) or pSUPER-p53(p53) were transfected with p2.1 reporter and pRL-SV40 (D). Cells were exposed to 20% and 1% O_2_ conditions with or without rhMIF treatment and then harvested for luciferase assay. Fold induction of relative luciferase activity was calculated. A normalized mean value±S.D. of three independent transfections is shown as fold induction. *; *p*<0.05 compared to respective control (ANOVA). (E) MCF-7 and MDA-MB-231 cells transfected with pFLAG-MIF-wt plasmid were exposed to 1% O_2_ and then cells were harvested. 500 µg of lysates were incubated with anti-FLAG affinity agarose beads and captured protein was eluted and analyzed by Western blot with anti-p53 antibody. 50 µg of lysate were analyzed by Western blot with anti-p53 antibody.

MIF was identified as a binding protein and a functional suppressor of p53 [28], [30]. We next explored a possibility that p53 is involved in MIF-induced HIF-1 activation. The effect of rhMIF on HIF-1α protein expression in MDA-MB-231 cells, which express mutant p53, and p53-null osteosarcoma Saos-2 cells was examined (Fig. 6A). In contrast to results with MCF-7 cells harboring wild-type p53, rhMIF treatment did not increase HIF-1α protein expression under 20% or 1% O_2_ in either MDA-MB-231 or Saos-2 cells (Fig. 6B). Moreover, rhMIF treatment did not enhance hypoxia-induced HRE-dependent gene expression in Saos-2 cells (Fig. 6C). In accord with this result, MCF-7 cells transfected with the vector encoding short-hairpin RNA against p53 [31] did not respond to rhMIF under hypoxic conditions (Fig. 6D), although basal HIF-1 activity was unregulated by knockdown of p53. To investigate involvement of wild-type p53 in regulation of HIF-1α protein, binding between MIF and p53 in MCF-7 and MDA-MB-231 cells was examined. The amount of captured p53 was less in MDA-MB-231 cells than MCF-7 cells, which expresses wild-type p53 (Fig. 6E).

## Discussion

In this study, we demonstrate that intracellular overexpression or extracellular administration of MIF increased HIF-1α protein expression and HIF-1-dependent gene expressions in MCF-7 breast cancer cells. Knockdown of MIF or CD74 expression by siRNA suppressed hypoxia-induced HIF-1α activation. In addition, we demonstrated that the effect of MIF on HIF-1 is observed only in the presence of wild-type not but mutant p53.

We adopted two protocols of MIF treatment. One is intracellular overexpression of MIF, and extracellular administration of bacterially produced rhMIF. Both the treatments enhanced hypoxia-induced HIF-1 activation. It is proposed that cells take up extracellular MIF is taken by endocytosis in membrane-bound vesicles, which interact with intracellular signaling molecules including Jab1/CSN5 [29] and p53 [32].

MIF interacts with the intracellular signaling intermediate Jab1/CSN5, which was reported to regulate the stability of HIF-1α protein [28], [29], [33]. Overexpression of Jab1 stabilizes HIF-1α protein in HEK293 and MIA-paca-2 cells and activates HIF-1-dependent transcription [28], [34]. But in MCF-7 cells, forced expression of Jab1 did not increase HIF-1α protein or HRE-dependent gene expression under either 20% or 1% O_2_. These results indicate that Jab1/CSN5 does not critically contribute to MIF-induced HIF-1 activation in MCF-7 cells. The molecular mechanism underlying the difference between cell lines remains to be established.

Several reports demonstrated that MIF is an oxidoreductase with insulin disulfide bond reducing activity [22]–[24]. As shown in Fig. 4, the redox mutant MIF-C57S/C60S can activate HIF-1. TRX is also an oxidoreductase that is involved in intracellular signal transduction [24]. In contrast to previous reports using MCF-7 cells [35], [36], but in accordance with our report using HeLa cells [25], neither overexpression nor knockdown of TRX affected HIF-1α protein expression at 20% O_2_ or 1% O_2_ in the present study (Fig. 3C and 4B). Moreover, treatment with antioxidants did not affect HIF-1α protein expression (Fig. 4C). Together, we conclude that MIF enhances HIF-1 activity in a redox activity-independent manner.

**Figure 7:**
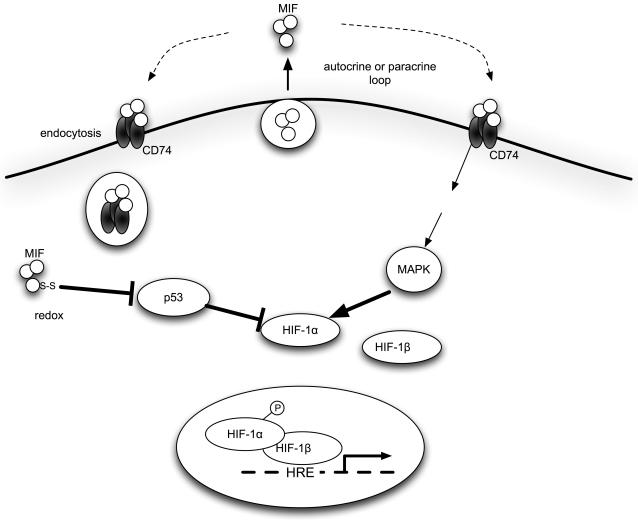
Involvement of MIF in HIF-1 regulation in MCF-7 cells. HIF-1α protein stabilization and transactivation activity induced by MIF is dependent on p53- and MAPK-dependent signaling pathway.

The type II transmembrane protein CD74 was identified as a high-affinity binding protein for MIF [26]. In this study, we indicated that knockdown of CD74 expression by siRNA made MCF-7 cells to be insensitive to MIF treatment in expression of HIF-1α under hypoxia (Fig. 4D). MIF binds to the extracellular domain of CD74, and CD74 is required for MIF-induced activation of the extracellular signal–regulated kinase–1/2 MAP kinase cascade, cell proliferation, and release of arachidonates such as PGE_2_ [37], [38]. We also showed that MIF-induced HIF-1α transcriptional activation in MCF-7 cells was blocked by the MEK inhibitors PD98059 or U0126 (Fig. 4E). The evidence indicates that CD74-dependent signaling pathway contributes to extracellularly administrated MIF-induced HIF-1 activation (Fig. 7).

MIF is a potent cytokine that overcomes p53 function by suppressing its transcriptional activity [30], [39]. It was reported that p53 interacts with HIF-1α *in vivo* and that p53 promotes the ubiquitin-mediated degradation of HIF-1α [40]. The experimental results shown as Fig. 6 indicates that effect of MIF on HIF-1 is p53-dependent and suggests that MIF stabilizes HIF-1α protein under hypoxic conditions by sequestrating p53 from HIF-1α (Fig. 7).

In addition to MIF's potent effects on the immune system, MIF is linked to fundamental processes conferring cell proliferation, cell survival, angiogenesis, and tumor invasiveness. A line of studies report the increased expression of MIF in solid tumors. Several studies also indicate that MIF expression closely correlates with tumor aggressiveness and metastatic potential, suggesting as important contribution to cancer malignancy by MIF [41]. The link of MIF to HIF-1α protein stabilization and transactivation activity provides molecular mechanism for MIF-dependent promotion of tumorigenesis.

## Materials and Methods

### Cell culture and reagents

Human breast carcinoma MCF-7 (wild-type p53), MDA-MB-231 (mutant p53), and osteosarcoma Saos-2 (p53-null) cell lines were grown in Dulbecco's modified essential medium supplemented with 10% FBS, 100 units/ml penicillin, and 100 g/ml streptomycin. *N*-acetyl-cysteine (NAC), the iron chelator desferrioxamine (DFX), and α-tocopherol were from Sigma-Aldrich (St. Louis, MO). Recombinant human (rh) MIF was from R&D systems (Minneapolis, MN). Recombinant human thioredoxin (TRX) was prepared following a protocol described previously [42], [43].

### Plasmids

Human MIF or Jun activation domain-binding protein 1(Jab1) cDNA was cloned by RT-PCR from a HeLa cell cDNA library, using specific primers to based on the human MIF or Jab1 sequence, respectively. For expression plasmids with N-terminal 3xFLAG-tagged MIF and TRX, PCR-amplified fragments were inserted into p3xFLAG-CMV-10 plasmid (Sigma-Aldrich). Mutagenesis of MIF (C57/60S) was performed using a protocol on pCMV-3xFLAG-MIF [44]. C32/35S-TRX mutant was described previously [43]. To establish stable transfectants, 3xFLAG-tagged expression cassettes of MIF and TRX were amplified by PCR and transferred to the expression vector pQCXIP (Clonthech, Palo Alto, CA). For the influenza hemagglutin (HA) epitope-tagged Jab1 expression plasmid pSRα-HA-Jab1, PCR-amplified cDNA of Jab1 was inserted into pSRα-HA1 [45]. A vector encoding short-hairpin RNA against p53 (pSUPER-p53) was kindly provided by Dr. Agami, the Netherlands Cancer Institute [31].

### Hypoxic treatment

Tissue culture dishes were transferred to a modular incubator chamber (Billups-Rothenberg, Del Mar, CA) which was flushed with 1% O_2_-5% CO_2_-94% N_2_, sealed, and placed at 37°C [46], [47].

### Immunoblot assays

Whole cell lysates were prepared using ice-cold lysis buffer [0.1% SDS, 1% NP40, 5 mM EDTA, 150 mM NaCl, 50 mM Tris-Cl (pH 8.0)] supplemented with7 2 mM dithiothreitol, 1 mM NaVO_3_, and Complete protease inhibitor™ (Roche Diagnostics, Basel, Switzerland). Samples were centrifuged at 10,000× *g* to pellet cell debris. For HIF-1α and HIF-1β, 100-µg aliquots were fractionated by SDS-polyacrylamide gel electrophoresis (SDS-PAGE) and subjected to immunoblot assay using mouse monoclonal antibodies against HIF-1α (BD Biosciences, San Jose, CA), or HIF-1β (BD Biosciences) at 1∶1000 dilution and HRP-conjugated sheep antibody against mouse IgG (GE Healthcare Bio-Science Corp., Piscataway, NJ.) at 1∶2000 dilution [46]. For MIF, TRX, Jab1, and p53, 20-µg aliquots were analyzed by SDS-PAGE and subjected to immunoblot assay using rabbit polyclonal antibodies against MIF (FL-115, Santa Cruz Biotechnology, Inc., Santa Cruz, CA), TRX (FL-105, Santa Cruz Biotechnology, Inc), Jab1 (FL-334, Santa Cruz Biotechnology, Inc.), and p53 (DO-1, Santa Cruz Biotechnology, Inc) at 1∶1000 dilution and HRP-conjugated goat antibody against rabbit IgG (GE Healthcare Bio-Science Corp.) at 1∶2000 dilution. Chemiluminescent signal was developed using ECL reagent (GE Healthcare Bio-Science Corp.).

### Reverse Transcription (RT)-PCR

The RT-PCR protocol was described previously [46], [47]. After treatment, cells were harvested and RNA was isolated with TRIzol™ (Invitrogen). 1 µg of total RNA was subjected to first strand cDNA synthesis using random hexamers (SuperScript II RT kit, Invitrogen). cDNAs were amplified with TaqGold™ polymerase (Roche Diagnostics) in a thermal cycler with the specific primers (sequences provided on request). For each primer pair, PCR was optimized for cycle number to obtain linearity between the amount of input RT product and output PCR product. Thermocycling conditions were 30 s at 94°C, 60 s at 57°C, and 30 s at 72°C for 28 (glucose transporter 1 [GLUT1]), 25 (VEGF and HIF-1α) and 22 (18S) cycles preceded by 10 min at 94°C. PCR products were fractionated by 1% SeaKem GTG Agarose gel electrophoresis, stained with ethidium bromide, and visualized with UV.

### Reporter Gene Assays

Reporter assays were performed in MCF-7 cells. 5×10^4^ cells were plated per well on the day before transfection. Plasmid p2.1 contains a 68-bp HRE from the ENO1 gene inserted upstream of an SV40 promoter in the luciferase reporter plasmid pGL2-Promoter (Promega, Madison, WI). Constructs encoding the GAL4 DNA-binding domain (amino acids 1–147) fused to the amino acids of HIF-1α (531–826) were analyzed for their ability to transactivate reporter gene GAL4E1bLuc, which contains 5 copies of a GAL4 binding site upstream of a TATA sequence and firefly luciferase coding sequences. The reporter gene plasmid and the control plasmid pRL-SV40 (Promega), which contains SV40 promoter sequences upstream of *Renilla reniformis* luciferase coding sequences, were pre-mixed and used. In each transfection, indicated dose of test plasmids, 200 ng of reporter gene plasmid, and 50 ng of the control were pre-mixed with Fugene 6™ transfection reagent (Roche Diagnostics). In each assay the total amount of DNA was held constant by addition of corresponding empty vectors. After treatment, the cells were harvested and the luciferase activity was determined using the Dual-Luciferase Reporter Assay System (Promega). The ratio of firefly to *Renilla* luciferase activity was determined. A normalized mean±S.D. based on three independent transfections is shown as relative luciferase activity (RLA).

### Gene silencing using short interfering RNA (siRNA)

Two oligonucleotides consisting of ribonucleosides except for the presence of 2′-deoxyribonucleosides (dTdT) at the 3′ end, 5′-GCCUGCACAGCAUCGGCAAdTdT-3′ and 5′-UUGCCGAUGCUGUGCAGGCdTdT-3′ for siRNA_MIF_ and 5′-CCAUCUGCGUGACAAUAAAdTdT-3′ and 5′-UUUAUUGUCACGCAGAUGGdTdT-3′ for siRNA_TRX_, were synthesized and annealed (Qiagen Inc., Valencia, CA.). siRNAs corresponding to human CD74 (cat. no. SI00063049) were from Qiagen Inc. MCF-7 cells were transfected by 100 nM siRNA using HiPerFect™ Transfection Reagent (Qiagen Inc.) following a protocol provided by the manufacturer.

### Data analysis

All the experiments were done at least three times (unless mentioned otherwise) and representative blots are shown. Data were expressed as mean±S.D. Significance tests (ANOVA with *post-hoc* test or Student's *t*-test) were performed using Prism™ version 4 application.
